# Supplementary material for: Nodal is a short-range morphogen with activity that spreads through a relay mechanism in human gastruloids
Source: Nat Commun. 2022 Jan 25;13:497. doi: 10.1038/s41467-022-28149-3 (PMC8789905; doi:10.1038/s41467-022-28149-3)
Supplement: Supplementary file 1 — Supplementary Information [file 41467_2022_28149_MOESM1_ESM.pdf]

Supplementary information

Nodal is a short-range morphogen with activity that spreads through a relay mechanism in human gastruloids.

Lizhong Liu[1], Anastasiia Nemashkalo[2], Luisa Rezende[1], Ji Yoon Jung[1], Sapna Chhabra[1,3], M. Cecilia Guerra[1], Idse Heemserk[4,5], Aryeh Warmflash[1,6,*]

Affiliations:[1]Department of Biosciences, Rice University; Houston, USA.

[2] Los Alamos National Laboratory, CINT/B11 Division; Los Alamos, USA.

[3]Developmental Biology Unit, EMBL Heidelberg; Heidelberg, Germany.

[4]Department of Cell and Developmental Biology, University of Michigan Medical School; Ann Arbor, USA.

[5]Department of Physics, University of Michigan; Ann Arbor, USA.

[6]Department of Bioengineering, Rice University; Houston, USA.

[*]Author for correspondence: aryeh.warmflash@rice.edu

Supplementary Figures

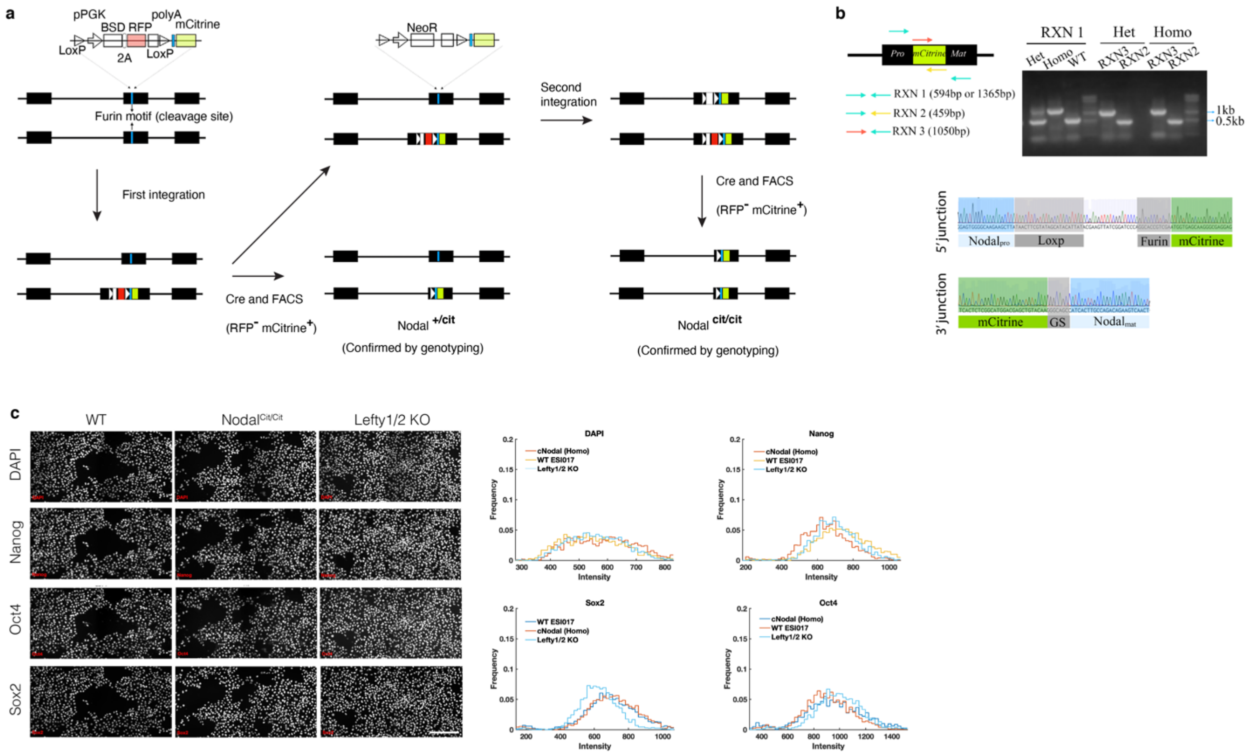

Supplementary Figure 1. Insertion of mCitrine to targeted Nodal locus and cNodal knock-in and Lefty compound knock-out hESCs are pluripotent. (a) Schematic of CRISPR/Cas9 mediated double-strand DNA break (DSB) repair by homologous recombination for engineering the Nodal locus. The cleavage site (blue vertical bar) is located within Exon2 of the *NODAL* gene, specifically, within the last codon of the 'RHRR' Furin consensus motif. Linearized donor DNA with 400-800 bp homology arms was provided for DSB repair. The donor DNA is composed of the fluorescent protein and loxP sites flanking an antibiotic resistance gene expression cassette, i.e., blasticidin S deaminase (BSD) or neomycin/kanamycin phosphotransferase (NeoR). mTagRFP was co-expressed with BSD to indicate chromosomal integration of donor DNA. On day 3 post-nucleofection, Blasticidin or G418 was added into the medium to select cells with integration. Resistant cells were amplified, and then transfected with Cre expression plasmid DNA to excise the fragment between loxP sites. After excision, Nodal open reading frame (ORF) is reconstituted with a loxP site and in-frame mCitrine. The residual loxP sequence is upstream of the Furin consensus motif and will be removed from the mCitrine::Nodal (mature) fusion by the convertase. Single cell FACS was performed to isolate successfully modified mono-clones. (b) Confirmation of integration. Three pairs of primers were used to genotype retrieved clones. Reaction (RXN) 1 with a pair of primers covers the entire integrated fragment, distinguishing modified allele from wide type allele via size difference. RNX2 and RNX3 amplify 5' and 3' adjacent regions, respectively. The entire gel is shown. The entire integrated fragment was sequenced, and the junctions between donor and genomic DNA are shown. 3 experiment was repeated independently with similar results. (c) Pluripotency markers Oct4, Sox2 and Nanog levels were checked via immunofluorescence staining and compared to

parental cells. WT, wild type ESI-017 cells. Nodal$^{cit/cit}$ (clone H3), ESI-017 cells with biallelic mCitrine knock-in to Nodal locus. Lefty1/2 KO, ESI-017 cells with lefty 1 and Lefty 2 compound knock-out. DAPI was used to stain nuclei, Oct4, Sox2, Nanog levels are quantified based on fluorescence intensity. 3 experiment was repeated independently with similar results. Scale bar, 100 µm.

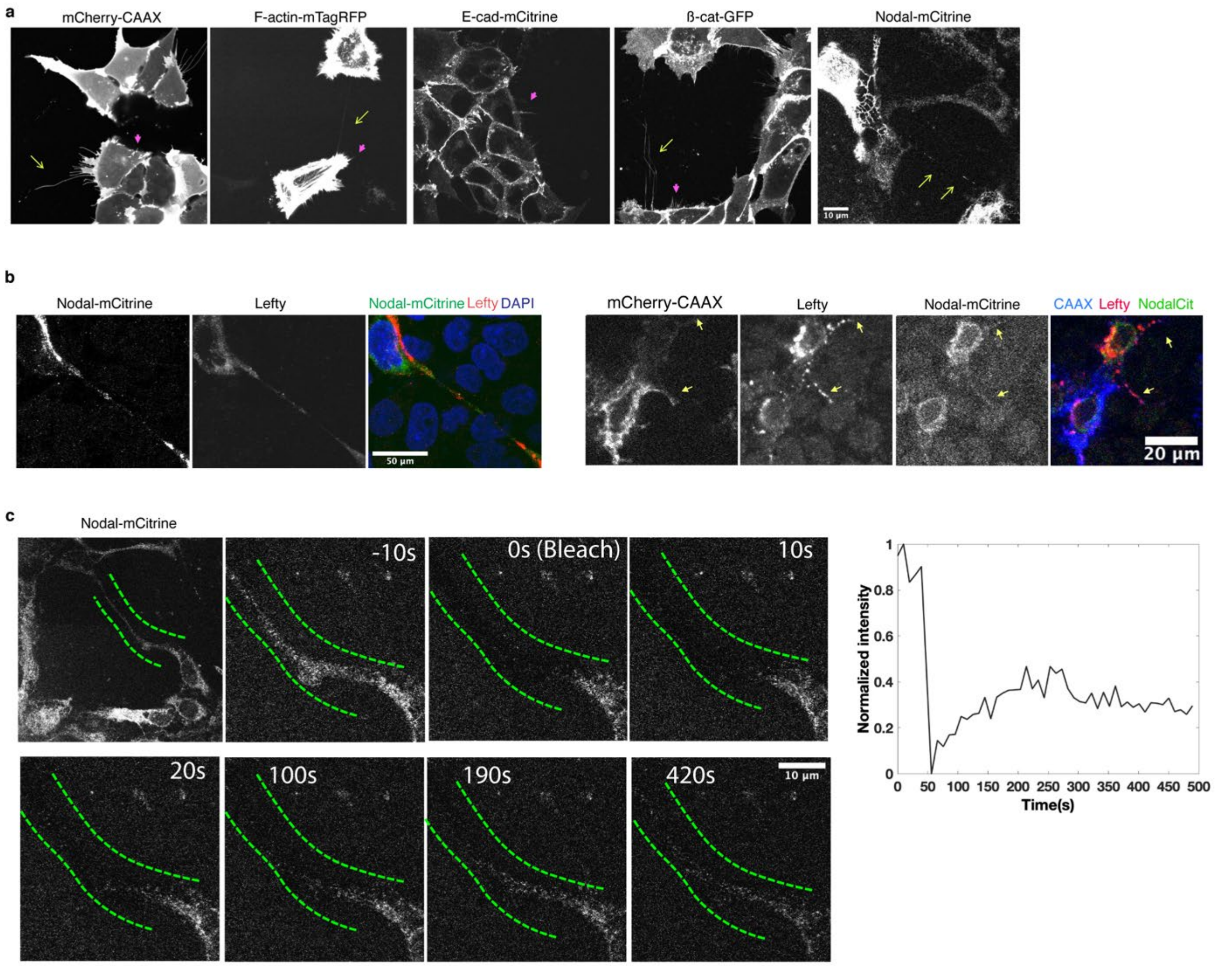

Supplementary Figure 2. Nodal and Lefty proteins are found in membrane protrusions. (a) High magnification of hESCs with mCherry labeled cell membrane, mTagRFP labeled F-actin, mCitrine labeled E-cadherin (cEcad), GFP labeled ß-catenin and mCitrine labeled Nodal (cNodal) shows thread-like structures and cNodal localized on those thread-like structures connecting two cells. Yellow arrowheads indicate long thread-like structure and cNodal puncta, pink arrowheads indicate short spikes. (b) Nodal and Lefty locate on CAAX labeled protrusions. Homozygous cNodal cells (mCherry-CAAX) were co-cultured with Lefty 1/2 compound knock-out cells and treated with Activin for 24h. cNodal (anti-GFP antibody) and Lefty (anti-Lefty) were examined via immunostaining. (c) Fluorescence recovery after photobleaching (FRAP) shows cNodal was transported through a membrane protrusion connecting two cells. Green dash lines flank and indicate the photobleached protrusion. (a-c) Similar results were obtained in more than 3 repeated experiments.

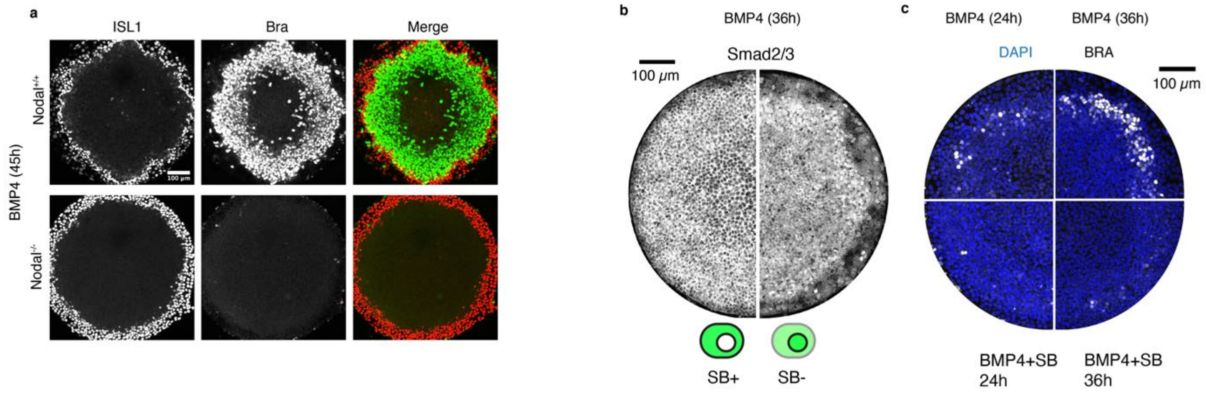

Supplementary Figure 3. Endogenous Nodal is required for mesodermal differentiation. (a) Wild type ESI-017 (Nodal$^{+/+}$) or homozygous Nodal knockout ESI-017 (Nodal$^{-/-}$) cells were used to make micropatterned gastruloids, respectively. Extraembryonic fate maker ISL1 and Mesodermal fate maker Brachyury (BRA) are shown. Scale bar, 100 μm. (b) Nodal signaling inhibitor SB-431542 inhibits Smad2/3 nuclear accumulation in cNodal 2D-gastruloids. (c) Nodal signaling is required for mesodermal differentiation. A mesodermal fate maker Brachyury (BRA) at 24h or 36h post-BMP4 treatment, with or without addition of Nodal signaling inhibitor SB-431542 in cNodal 2D-gastruloids. (a-c) Similar results were obtained in more than 3 repeated experiments, Scale bar, 100 μm.

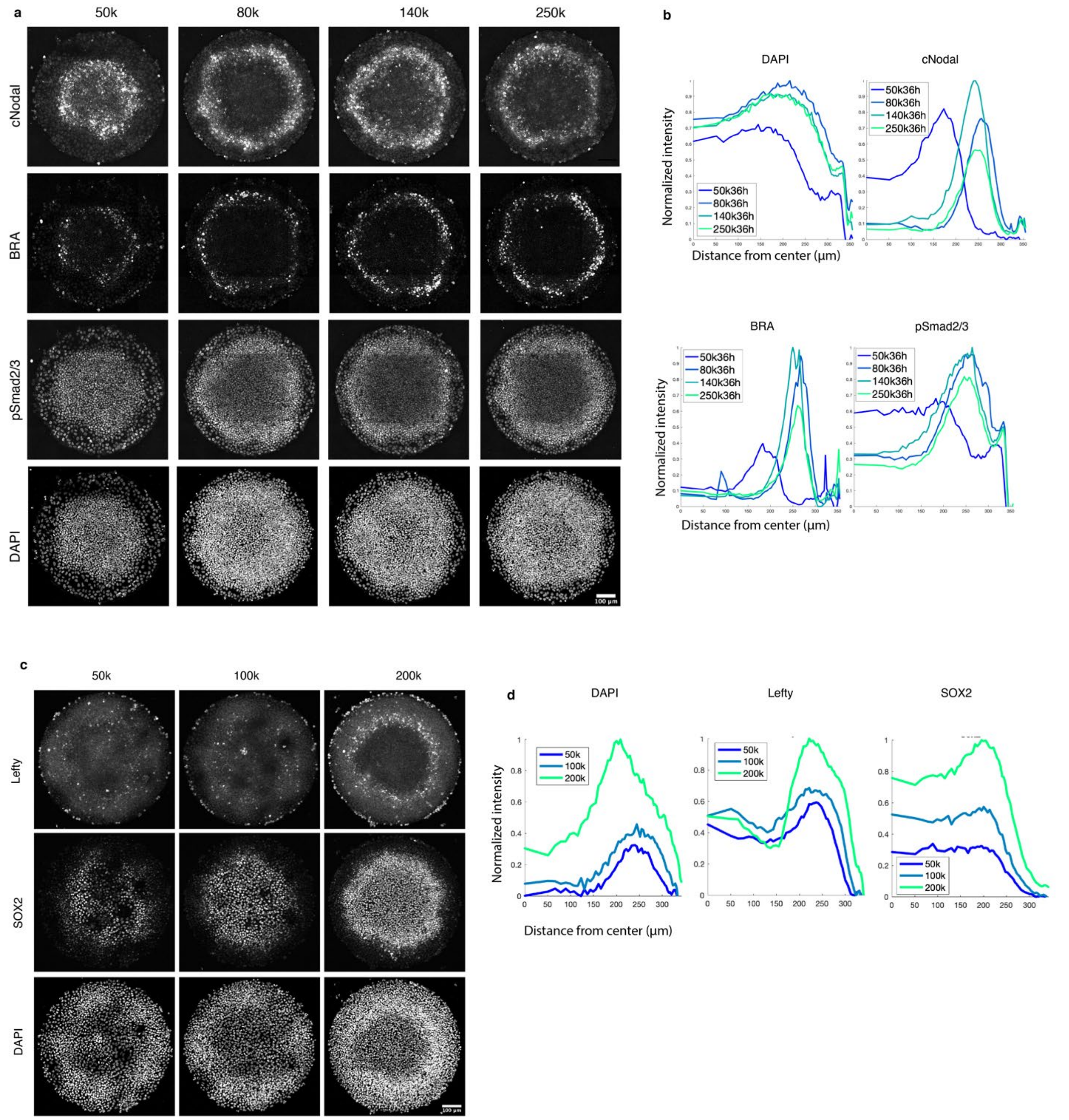

Supplementary Figure 4. Initial seeding cell density effects on Nodal and Lefty distribution in micropatterned gastruloids. (a) Representative images of cNodal and pSmad2/3 distribution at different initial cell density conditions. 50,000, 80,000, 140,000, or 250,000 cells were initially seeded in each well of 96-well micropattern plate, respectively. Gastruloids were fixed at about 39h post-BMP4 induction. (b) cNodal and pSmad2/3 levels were quantified as function of distance from colony center (n=6). (c) Representative images of Lefty protein and ectoderm/pluripotency marker Sox2 distribution at different initial cell densities. 50,000, 100,000, or 220,000 cells were initially seeded in each well of 96-well micropattern plate. Gastruloids were fixed at about39h post-BMP4 induction. (d) Lefty and Sox2 levels were quantified as function of distance from colony center (n=3).

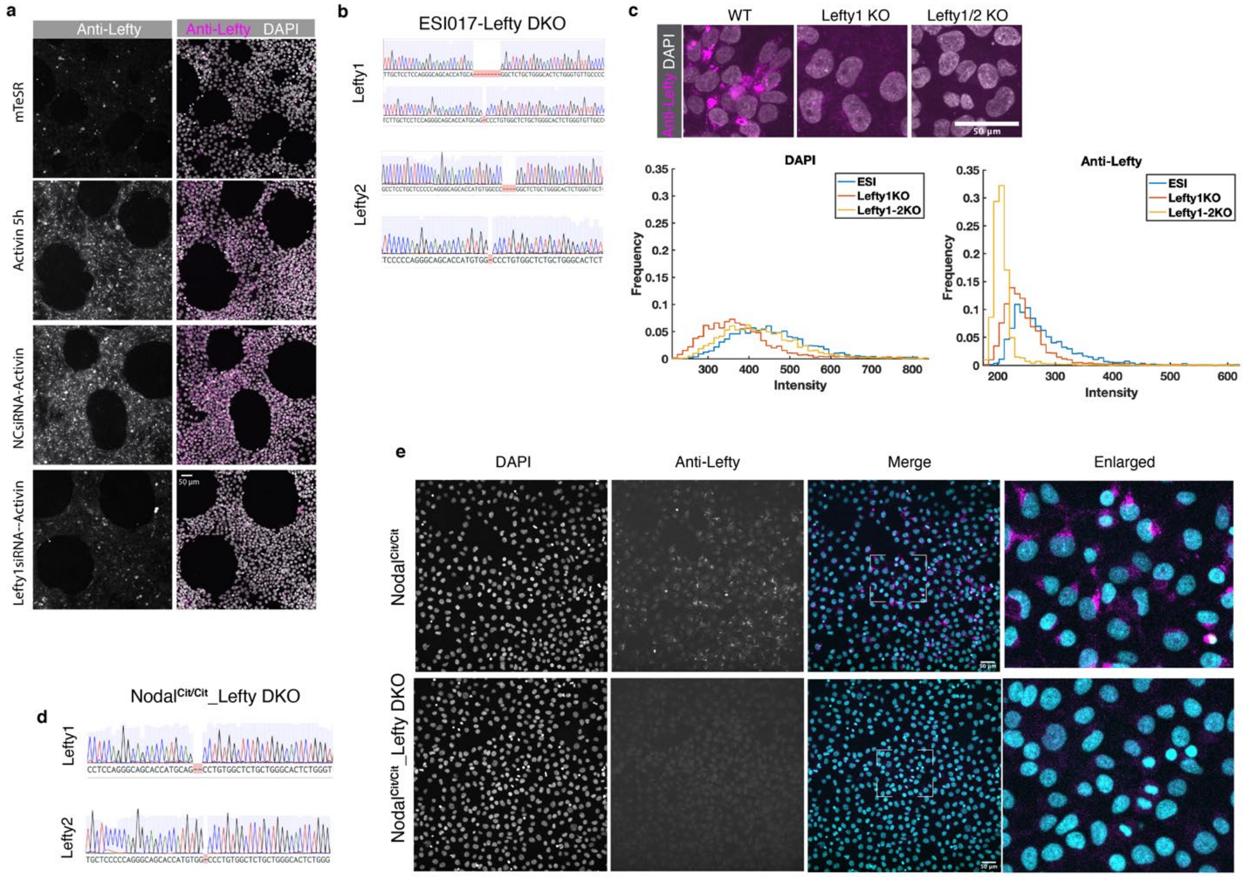

Supplementary Figure 5. Validation of Lefty1/2 knock-out. (a) Lefty knock down experiments show that Lefty antibody staining quantitatively reflects the changing of Lefty expression levels, scale bar, 50 µm. (b) Lefty 1/2 compound knock-out was verified by sequencing. Deletions causing open reading frame (ORF) shifts were found closely down-stream of the start codons of both alleles of the Lefty 1 and Lefty 2 genes. (c) Immunostaining using an antibody that recognizes both Lefty 1 and Lefty 2 confirmed ablation of Lefty proteins. (top) Representative images of ESI-017 cells, Lefty1 single knock-out or Lefty 1/2 compound knock-out cells treated with Activin and fixed, scale bar, 50 µm. (bottom) Quantification of fluorescence intensity. (d) A clone of Nodal$^{Cit/Cit}$ cells with mutations in Lefty 1 and Lefty 2 gene was verified by sequencing of the PCR amplified products of the Lefty1 and Lefty2 loci. (e) Immunostaining using Lefty antibody, verified no expression of Lefty in Nodal$^{Cit/Cit}$-Lefty DKO cells induced with Activin. Representative images are shown, n=4 biological replicates, 3 experiments were repeated with similar results. Scale bar, 50 µm.

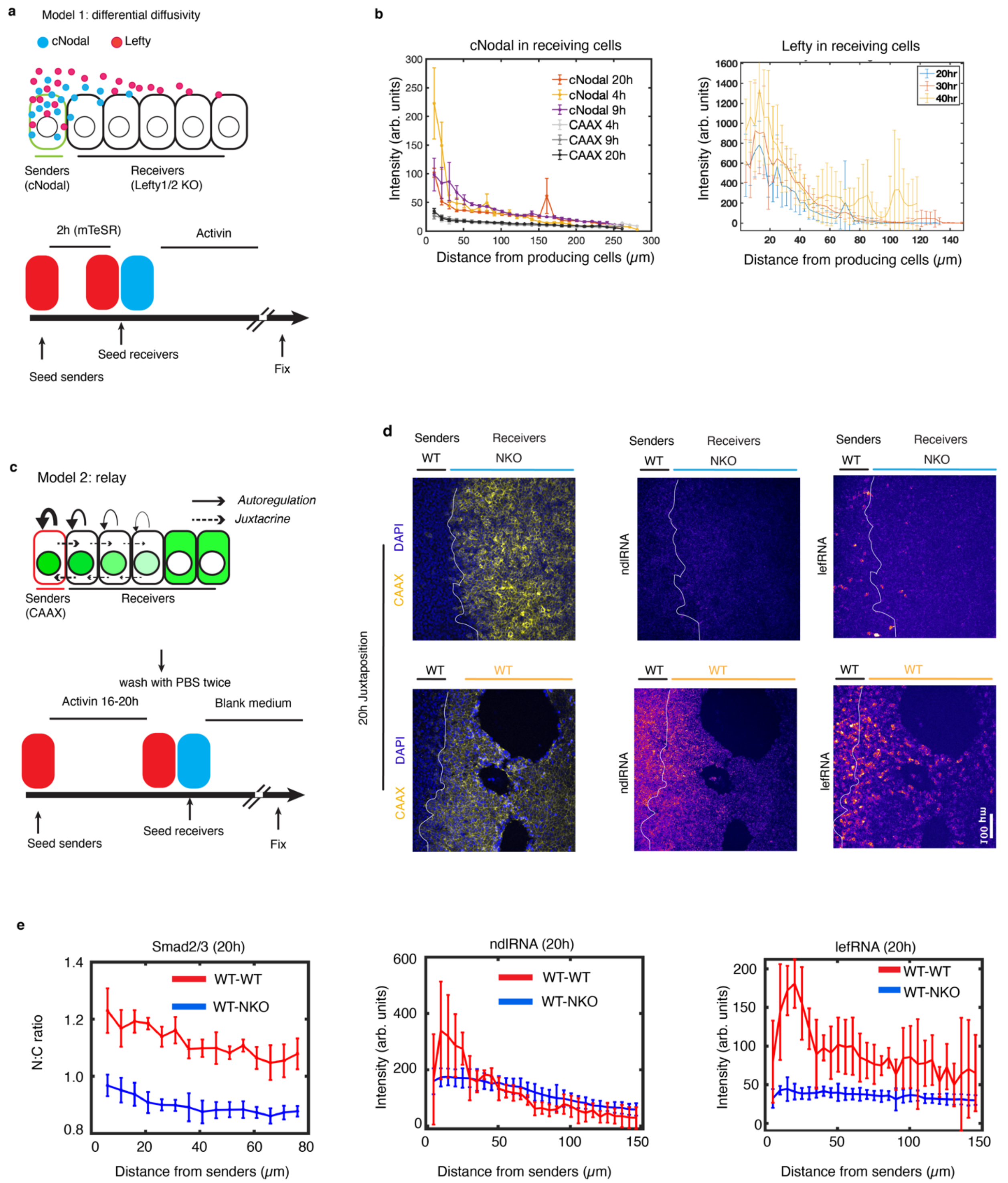

Supplementary Figure 6. Lefty protein is biochemically able to diffuse over several cell diameters while Nodal protein only reaches to immediately adjacent cells. (a) Schematic illustration of juxtaposition experiments for protein differential diffusivity model, also shown in Fig. 2. While sender cells produce and secrete cNodal and Lefty proteins, receiving cells cannot make either cNodal or Lefty. In particular, sender cells (homozygous cNodal cells with mCherry-CAAX membrane label), were seeded in a well of culture insert and incubated with ROCK-inhibitor for 2h allowing the cells to attach. The receiver Lefty1/2 compound knock-out cells were then seeded adjacent to sender

cells, and 10 ng/ml Activin was added to induce expression of cNodal and Lefty. (b) Cells were fixed at various time points post-juxtaposition. Lefty and cNodal levels in receiving cells were examined through immunofluorescence staining, and fluorescence intensity was quantified as function of distance from the border with producing cells at the indicated times. mCherry-CAAX that labels cell membrane of sender cells was also quantified as a control. Data are presented as mean values +/- SEM, n=4 biological replicates. (c) Schematic of relay model, also shown in Fig. 3. Senders represent induced cells that express Nodal. Solid line with arrowhead, represents Nodal positive autoregulation. Dashed lines with arrowheads represent juxtacrine induction by Nodal. Black solid circle represents the cell nucleus, green color represents Smad2/3. (d-e) Experimental test of the relay model. (d) Nodal and Lefty RNA FISH to measure Nodal and Lefty transcripts in juxtaposition experiments with cells with the indicated genotypes. Receiver cells in all conditions were labelled by mCherry-CAAX. (e) Experiments performed as in (d) and Nodal mRNA, Lefty mRNA and Smad2/3 were measured. Smad2/3 nuclear to cytoplasmic ratio, Nodal RNA and Lefty RNA are quantified as a function of distance from border. Data are presented as mean values +/- SEM, n=4 biological replicates for each condition. Smad2/3 was measured in a separate experiment from Nodal and Lefty mRNA.

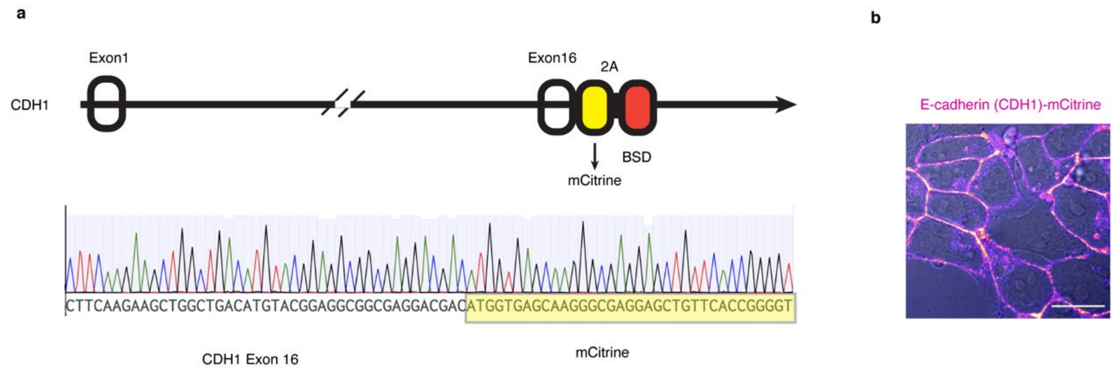

Supplementary Figure 7. Insertion of mCitrine to CDH1 (E-cadherin) cytoplasmic C-terminal. (a) Sequencing result of modified CDH1 locus showing connection of CDH1 Exon 16 and mCitrine coding sequence. (b) A representative image of E-cadherin-mCitrine fusion localized on the cell membrane. Similar results were obtained in more than 3 repeated experiments. Scale bar, 20 µm.

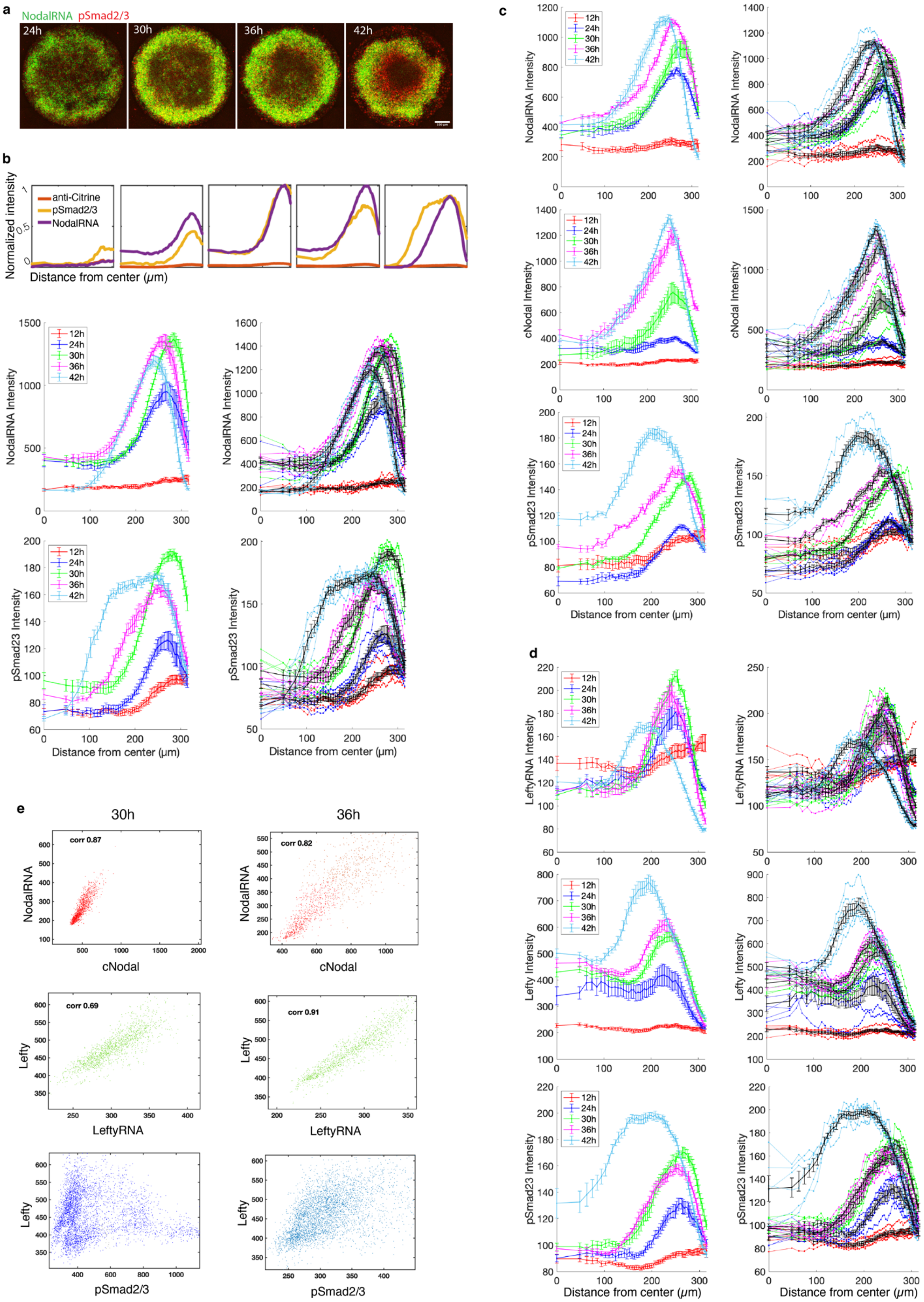
a
NodalRNA pSmad2/3
24h
30h
36h
42h
b
Normalized intensity
anti-Citrine
pSmad2/3
NodalRNA
Distance from center (μm)
12h
24h
30h
36h
42h
NodalRNA Intensity
pSmad23 Intensity
Distance from center (μm)
c
NodalRNA Intensity
cNodal Intensity
pSmad23 Intensity
Distance from center (μm)
d
LeftyRNA Intensity
Lefty Intensity
pSmad23 Intensity
Distance from center (μm)
e
30h
36h
corr 0.87
corr 0.82
NodalRNA
cNodal
corr 0.69
corr 0.91
Lefty
LeftyRNA
Lefty
pSmad2/3

Supplementary Figure 8. Nodal and Lefty show limited diffusion from source cells in 2D gastruloids. (a) Representative images of Nodal smFISH (green) and pSmad2/3 (red) at 42h of Nodal$^{+/+}$ without mCitrine tagging (WT). Scale bar 100 µm. (b) Simultaneous quantification of Nodal RNA and pSmad2/3 in WT cells. (top) Mean intensity is quantified over time as function of distance from colony center. (bottom) Curves of individual colonies are also shown. Data are presented at mean, n=6 biological replicates. (c) Curves of individual colonies for data shown in Fig 4d. Data are presented as mean values +/- SEM, n=6 biological replicates. (d) Curves of individual colonies for data shown in Fig 4f. Data are presented as mean values +/- SEM, n=6 biological replicates. Arbitrary units are used in b, c, d. (e) mRNA and protein of cNodal, Lefty, and pSmad2/3 in gastruloids at 30h and 36h were examined. The correlation of cNodal protein and mRNA (left-most panels), Lefty protein and mRNA (middle panels) levels in single cells were analyzed, correlation coefficient is shown. The relationship between Lefty protein and pSmad2/3 (right panels) was also analyzed.

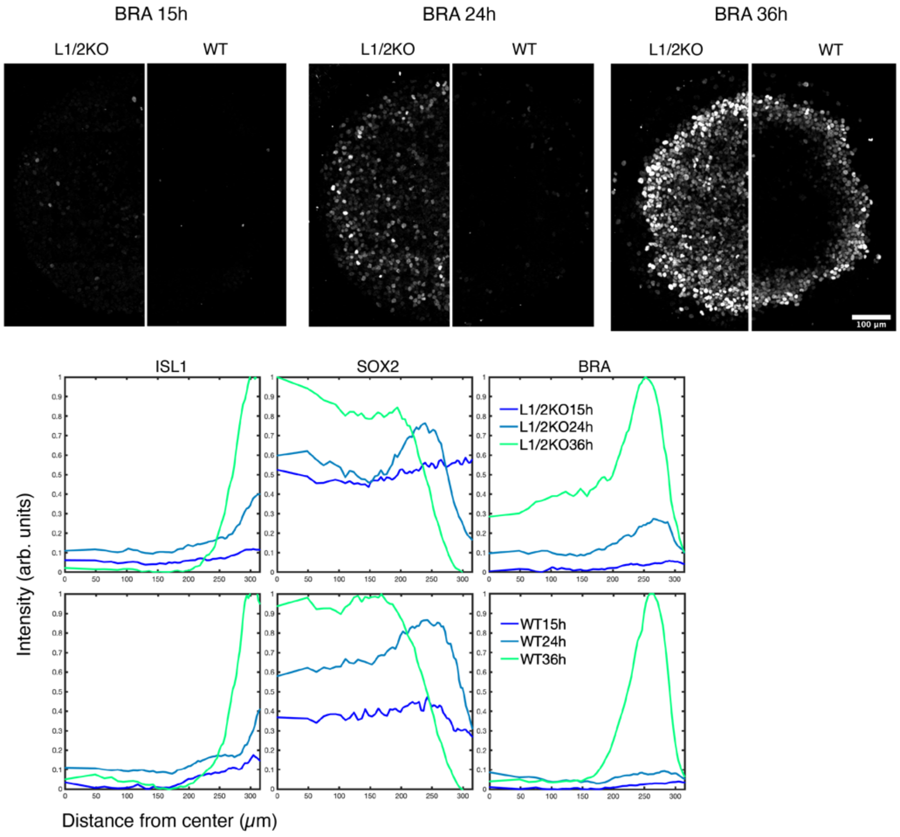

Supplementary Figure 9. Lefty1/2 knock-out causes earlier and broader expression of mesodermal marker Brachyury. Gastruloids created with wild type or Lefty 1/2 compound knock-out cells were fixed at 15h, 24h, or 36h, as indicated. Extraembryonic marker ISL1, mesodermal marker Brachyury (BRA), and ectodermal/pluripotency marker Sox2 were examined by immunofluorescence staining. Representative images of BRA at indicated time points are shown in upper panel, mean intensity of ISL1, Sox2 and BRA as function of distance from colony center is shown in lower panel (n=6). Scale bar, 100 µm.

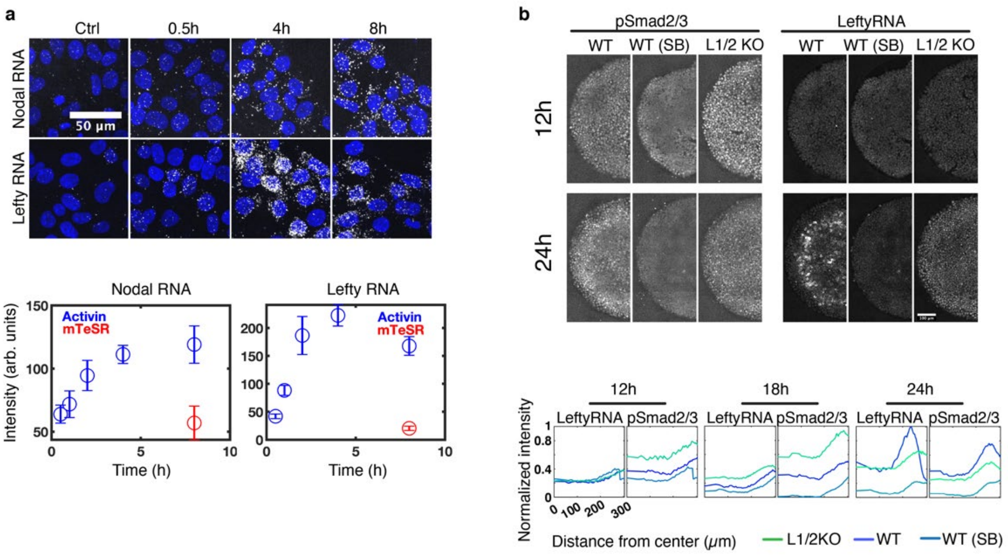

Supplementary Figure 10. smFISH shows dynamics of Lefty transcription in standard culture and gastruloids (a-b) smFISH of Nodal and Lefty RNA. Cells were treated with Activin for 0.5h, 4h, 8h or no treatment for 8h. (A) Representative images of Nodal smFISH (upper panel), Lefty smFISH (lower panel), nuclei were indicated by DAPI (blue), scale bar 50 µm. (b) quantification of smFISH signal in each nucleus, mean value of four images for each condition is shown, SEM is shown. (c-d) Lefty transcription and pSmad2/3 in WT or Lefty1/2 KO gastruloids. (c) Representative images of Lefty smFISH and pSmad2/3 staining at 12h and 24h of gastruloids made with WT hESCs, Lefty1/2 compound knockout (L1/2 KO) cells or WT hESCs in the presence of SB-431542 are shown. (d) Normalized mean intensity of signal of interest at 12h, 18h and 24h was quantified as function of distance from center, n=6. Scale bar, 100 µm.

Supplementary Table 1 Key reagent or resource

Reagent	Dilution / concentration	Source	Identifier
Mouse anti-GFP	1:200	Abcam	Cat# ab1218
Mouse anti-Smad2/3	1:200	BD Biosciences	Cat# 610843
Mouse anti-ISL1	1:75	DSHB	Cat# 39.4D5
Mouse anti-Oct3/4	1:400	BD Biosciences	Cat# 611203
Rabbit anti Eomes	1:200	Abcam	Cat# ab23345
Rabbit anti-pSmad1/5/8	1:100	Cell Signaling	Cat# 13820
Rabbit anti-pSmad2/3	1:100	Cell Signaling	Cat# 8828S
Rabbit anti-Smad2	1:200	Cell Signaling	Cat# 5339S
Rabbit anti-SOX2	1:200	Cell Signaling	Cat# 5024S
Goat anti-BRA	1:300	R&D Systems	Cat# AF2085
Goat anti-HAND1	1:200	Cell Signaling	Cat# AF3168
Goat anti-Nanog	1:200	R&D Systems	Cat# AF1997
Goat anti-Lefty	1:200	R&D Systems	Cat# AF746
Goat anti-SOX17	1:200	R&D Systems	Cat# AF1924
Activin A	10-100 ng/ml	R&D Systems	Cat# 338-AC
BMP4	50 ng/ml	Fisher Scientific	Cat# 314BP050
Wnt3A	200-300 ng/ml	R&D Systems	Cat# 5036-WN
SB431542	10 µM	Stemgent	Cat# 04-0010-05
Nodal RNAscope probe	1x	ACD Bio	Cat# 416541-C3
Lefty RNAscope probe	1x	ACD Bio	Cat# 415651
Axin2 RNAscope probe	1x	ACD Bio	Cat# 400241-C3
Lefty1 siRNA	N/A	Dharmacon	Cat# L-013114-00-0005
Lefty2 siRNA	N/A	Horizon Discovery	L-017473-00-0005

Supplementary Table 2 Primers used for genomic DNA PCR

Target gene	Forward	Reverse
Nodal	5' GCATGGTTTTGGAGGTGACCAG 3'	5' ACATGCCTGGTACCTAGCACAG 3'
Nodal	5' ATGCTCTACTCCAACCTCTCG 3'	5' ATGTATGCATGGTTGGTC 3'
Lefty1	5' CAGCTGGGGGAACTGAGCCTCT 3'	5' TCAGCCTCCCACAGACCTCTGC 3'
Lefty1	5' AGACCACTGCCCTCCAGTG 3'	5' AACACCAGCAGGTGTGTGC 3'
Lefty2	5' ATTGTCAAGCTGCTGACAGC 3'	5' AGCAGGACTACATACTGGGC 3'
CDH1	5'TAGAATCTGAAAGCGGCTGATACTG3'	5'AACACAAACTTATTAGAGCTATAAAGTGG3'

Movie legends

Supplementary Movie 1. Activin treated micropatterned Nodal$^{Cit/Cit}$ cells with CFP-H2B nuclear marker were imaged for 43h with 30-minute interval. Scale bar, 100 µm. Maximum intensity projection is shown.

Supplementary Movie 2. Wnt3A treated micropatterned Nodal$^{Cit/Cit}$ cells with CFP-H2B nuclear marker were imaged for 43h with 30-minute interval. Scale bar, 100 µm. Maximum intensity projection is shown.

Supplementary Movie 3. BMP4 treated micropatterned Nodal$^{Cit/Cit}$ cells with CFP-H2B nuclear marker were imaged for 43h with 30-minute interval. Scale bar, 100 µm. Maximum intensity projection is shown.
