# Supplementary material for: Nodal is a short-range morphogen with activity that spreads through a relay mechanism in human gastruloids
Source: Nat Commun. 2022 Jan 25;13:497. doi: 10.1038/s41467-022-28149-3 (PMC8789905; doi:10.1038/s41467-022-28149-3)
Supplement: Supplementary file 3 — Description of Additional Supplementary Files [file 41467_2022_28149_MOESM3_ESM.pdf]

Description of Additional Supplementary Files

File name: Supplementary Movie 1

Description: Activin treated micropatterned $Nodal^{Cit/Cit}$ cells with CFP-H2B nuclear marker were imaged for 43h with 30-minute interval. Scale bar, 100 µm. Maximum intensity projection is shown.

File name: Supplementary Movie 2

Description: Wnt3A treated micropatterned $Nodal^{Cit/Cit}$ cells with CFP-H2B nuclear marker were imaged for 43h with 30-minute interval. Scale bar, 100 µm. Maximum intensity projection is shown.

File name: Supplementary Movie 3

Description: BMP4 treated micropatterned $Nodal^{Cit/Cit}$ cells with CFP-H2B nuclear marker were imaged for 43h with 30-minute interval. Scale bar, 100 µm. Maximum intensity projection is shown.
