# Supplementary material for: Nodal is a short-range morphogen with activity that spreads through a relay mechanism in human gastruloids
Source: Nat Commun. 2022 Jan 25;13:497. doi: 10.1038/s41467-022-28149-3 (PMC8789905; doi:10.1038/s41467-022-28149-3)
Supplement: Supplementary file 7 — Reporting Summary [file 41467_2022_28149_MOESM7_ESM.pdf]

Corresponding author(s): Aryeh Warmflash
Last updated by author(s): Jul 16, 2021

Reporting Summary

Nature Portfolio wishes to improve the reproducibility of the work that we publish. This form provides structure for consistency and transparency in reporting. For further information on Nature Portfolio policies, see our Editorial Policies and the Editorial Policy Checklist.

Statistics

For all statistical analyses, confirm that the following items are present in the figure legend, table legend, main text, or Methods section.

n/a	Confirmed	
☐	☒	The exact sample size (*n*) for each experimental group/condition, given as a discrete number and unit of measurement
☐	☒	A statement on whether measurements were taken from distinct samples or whether the same sample was measured repeatedly
☐	☒	The statistical test(s) used AND whether they are one- or two-sided<br>*Only common tests should be described solely by name; describe more complex techniques in the Methods section.*
☒	☐	A description of all covariates tested
☒	☐	A description of any assumptions or corrections, such as tests of normality and adjustment for multiple comparisons
☐	☒	A full description of the statistical parameters including central tendency (e.g. means) or other basic estimates (e.g. regression coefficient) AND variation (e.g. standard deviation) or associated estimates of uncertainty (e.g. confidence intervals)
☒	☐	For null hypothesis testing, the test statistic (e.g. *F*, *t*, *r*) with confidence intervals, effect sizes, degrees of freedom and *P* value noted<br>*Give P values as exact values whenever suitable.*
☒	☐	For Bayesian analysis, information on the choice of priors and Markov chain Monte Carlo settings
☒	☐	For hierarchical and complex designs, identification of the appropriate level for tests and full reporting of outcomes
☒	☐	Estimates of effect sizes (e.g. Cohen's *d*, Pearson's *r*), indicating how they were calculated

Our web collection on statistics for biologists contains articles on many of the points above.

Software and code

Policy information about availability of computer code

Data collection	Microscopy images shown in Fig. 1a, 2d, e, Supplementary Fig. S2, 3a, Fig. S7b were collected through Laser Scanning Confocal microscope with Fluoview software FV10-ASW4.2. Microscopy images shown in Fig. 1c-e, Fig. 2c, Fig. 3b-c, Fig.4 a, b, d, Fig. 5, Supplementary Fig. S3 b, c, Fig. S4, Fig. S5 a, b, Fig. S6 d, Fig. S8 a, Fig. S9 a, Fig. S10 a, b were collected through Spinning Disc confocal microscope with Andor software (Andor iQ3). Microscopy images shown in Supplementary Fig. S1 c were collected through Epi-Fluorescence microscope with CellSens Dimension 1.18. Benchling (https://www.benchling.com/crispr/; also available at crispr.mit.edu/benchling) was used for guide design.
Data analysis	MATLAB (R2020b) for image processing, plots, kymographs and statistical analysis (e.g. Fig. 1, a-d), ilastik (1.3.3post3) for image segmentation mask. FIJI (ImageJ, 2.1.011.53) for lookup table.<br>Custom code for data analysis is available at https://github.com/warmflashlab/Liu2021.

For manuscripts utilizing custom algorithms or software that are central to the research but not yet described in published literature, software must be made available to editors and reviewers. We strongly encourage code deposition in a community repository (e.g. GitHub). See the Nature Portfolio guidelines for submitting code & software for further information.

Data

Policy information about availability of data

All manuscripts must include a data availability statement. This statement should provide the following information, where applicable:

- Accession codes, unique identifiers, or web links for publicly available datasets
- A description of any restrictions on data availability
- For clinical datasets or third party data, please ensure that the statement adheres to our policy

Source Data are provided with this paper. Data for individual micropattern colonies demonstrating variability in intensity are provided as Supplementary Figures.

Immunofluorescence and live-cell imaging raw data underlying quantification are provided (https://doi.org/10.6084/m9.figshare.17420450.v1). Plasmid DNA used in this study will be available from the corresponding author upon reasonable request.

Field-specific reporting

Please select the one below that is the best fit for your research. If you are not sure, read the appropriate sections before making your selection.

☒ Life sciences ☐ Behavioural & social sciences ☐ Ecological, evolutionary & environmental sciences

For a reference copy of the document with all sections, see nature.com/documents/nr-reporting-summary-flat.pdf

Life sciences study design

All studies must disclose on these points even when the disclosure is negative.

Sample size	The sample size was not predetermined for any of the experiments conducted in this study. Micropattern microscopy data were based on 6-10 biological replicates. For other immunostaining and smFISH data collected from standard cell culture, at least four replicates were measured. The sample size, based on previous experience, was sufficient as the experimental system (method) is highly reproducible between all replicates, which has been shown by our previous study (A. Warmflash et al. Nature Method 2014) and published works from multiple groups (F. Etoc et al. Developmental Cell 2016; I. Martyn et al. 2018 Nature; K. Minn et al. eLife 2020).
Data exclusions	No data was excluded from the analysis.
Replication	Experiments were repeated at least three times, and all attempts at replication were successful.
Randomization	To generate gastruloids, hESCs were dissociated, counted, and randomly allocated into each well of a CYTOO 96-well micropattern plates or ibidi chamber slides. For data acquisition, the region for imaging was randomly chosen within each group. For experiments with genetically modified cells, random allocation is not relevant, for example, the investigators need to know specific genotype and seed cells with different genotypes into separate wells, therefore random allocation was not performed for those experiments.
Blinding	To perform experiments, the investigators need to know the information regarding genotype and culture conditions, and therefore were not blinded to this information. All data analysis was performed with automated code and was therefore effectively blinded.

Reporting for specific materials, systems and methods

We require information from authors about some types of materials, experimental systems and methods used in many studies. Here, indicate whether each material, system or method listed is relevant to your study. If you are not sure if a list item applies to your research, read the appropriate section before selecting a response.

Materials & experimental systems

n/a	Involved in the study	
☐	☒	Antibodies
☐	☒	Eukaryotic cell lines
☒	☐	Palaeontology and archaeology
☒	☐	Animals and other organisms
☒	☐	Human research participants
☒	☐	Clinical data
☒	☐	Dual use research of concern

Methods

n/a	Involved in the study	
☒	☐	ChIP-seq
☒	☐	Flow cytometry
☒	☐	MRI-based neuroimaging

Antibodies

Antibodies used	mouse anti-GFP (Abcam, Cat#ab1218, 1:200), mouse anti-Smad2/3 (BD Biosciences, Cat# 610843, 1:200), mouse anti-ISL1 (DSHB, Cat#39.4D5, 1:75), mouse anti-Oct3/4 (BD Biosciences, Cat# 611203, 1:400), rabbit anti-Eomes (Abcam, Cat#ab23345, 1:200), rabbit anti-pSmad1/5/8 (Cell Signaling, Cat#13820, 1:100) rabbit anti-pSmad2/3 (Cell Signaling, Cat#8828S), rabbit anti-Smad2 (Cell Signaling, Cat# 5339S, 1:200), rabbit anti-Sox2 (Cell Signaling, Cat#5024S, 1:200), goat anti-Brachyury (R&D Systems, Cat#AF2085, 1:300), goat anti-Hand1 (R&D Systems, Cat# AF3168, 1:200), goat anti-Nanog (R&D Systems, Cat# AF1997, 1:200), goat anti-Lefty (R&D Systems, Cat# AF746, 1:200), goat anti-Sox17 (R&D Systems, Cat# AF1924, 1:200).
Validation	mouse anti-GFP (https://www.abcam.com/gfp-antibody-9f9f9-ab1218.html)<br>mouse anti-Smad2/3 (https://www.bdbiosciences.com/en-us/products/reagents/microscopy-imaging-reagents/immunofluorescence-reagents/purified-mouse-anti-smad2-3.610843)<br>mouse anti-ISL1 (https://dshb.biology.uiowa.edu/39-4D5)<br>mouse anti-Oct3/4 (https://www.bdbiosciences.com/en-us/products/reagents/microscopy-imaging-reagents/immunofluorescence-reagents/purified-mouse-anti-oct3-4.611203)<br>rabbit anti-Eomes (https://www.abcam.com/tbr2--eomes-antibody-ab23345.html)<br>rabbit anti-pSmad1/5/8 (https://www.cellsignal.com/products/primary-antibodies/phospho-smad1-ser463-465-smad5-ser463-465-

smad9-ser465-467-d5b10-rabbit-mab/13820)
rabbit anti-pSmad2/3 (https://www.cellsignal.com/products/primary-antibodies/phospho-smad2-ser465-467-smad3-ser423-425-d27f4-rabbit-mab/8828)
rabbit anti-Smad2 (https://www.cellsignal.com/products/primary-antibodies/smad2-d43b4-xp-rabbit-mab/5339)
rabbit anti-Sox2 (https://www.cellsignal.com/products/primary-antibodies/sox2-d6d9-xp-rabbit-mab-chip-formulated/5024)
goat anti-Brachyury (https://www.rndsystems.com/products/human-mouse-brachyury-antibody_af2085)
goat anti-Hand1 (https://www.rndsystems.com/products/human-hand1-antibody_af3168)
goat anti-Nanog (https://www.rndsystems.com/products/human-nanog-antibody_af1997)
goat anti-Lefty (https://www.rndsystems.com/products/human-mouse-lefty-antibody_af746)
goat anti-Sox17 (https://www.rndsystems.com/products/human-sox17-antibody_af1924)

Eukaryotic cell lines

Policy information about cell lines

Cell line source(s)	ESI-017 Human Embryonic Stem Cell line was purchased from ESI BIO, BioTime, Inc.
Authentication	Authentication was performed by checking cellular morphology, pluripotency markers (e.g. Sox2, Oct4, Nanog), and induced differentiation ability.
Mycoplasma contamination	Cells regularly tested negative for mycoplasma contamination.
Commonly misidentified lines (See ICLAC register)	None
